# Joint replacement surgery in Ghana (West Africa)—an observational study

**DOI:** 10.1007/s00264-019-04286-1

**Published:** 2019-01-24

**Authors:** Akintunde George, Paul Ofori-Atta

**Affiliations:** 1 Tissue Engineering Unit, Department of Chemical Engineering, University of Bath, Bath, UK; 2 West Hertfordshire NHS Trust, St. Joseph’s Orthopaedic Hospital, Koforidua, Ghana

**Keywords:** Hip and knee replacement surgery, Arthroplasty procedures, Arthroplasty registries

## Abstract

**Introduction:**

With the continued and effective transfer of orthopaedic knowledge and skills across continents, the incidence of hip and knee replacement surgery has increased in the developing world. More patients are having these procedures done locally rather than having to travel over to the more developed western countries at great financial costs for those who cannot really afford it. We report the data collected by an orthopaedic charity MOTEC LIFE UK which has been offering hip and knee arthroplasty procedures to patients who require them. The time period of the procedures was February 2013–October 2017.

**Methods:**

The data was collated prospectively and this included age, sex, indication for procedure, and side of procedure (including if bilateral). The information on hip implants used were also collected—implant type (cemented or uncemented), type of bearing surface, size of acetabular cup, liner, femoral head size, and stem size and including if screws were used to augment the fixation of the femoral cup. For the knee implants used, information on the type of implant (semi-constrained, cruciate retaining, or Stanmore hinge knee prosthesis), femoral and tibia stem size, insert type (fixed bearing or mobile bearing), and size; patella button size (whether patella replacement or circumcision) were collected. The surgical approach used in both knee and hip arthroplasty cases was noted.

**Results:**

It is seen from the data collected that a total of 113 hip arthroplasty procedures from 109 patients were carried out and for total knee replacements, 82 knee arthroplasty procedures from 76 patients were carried out. The above procedures were carried out in two separate hospitals. Degenerative osteoarthritis still remains the main indication for hip and knee arthroplasty surgery in our study and this is similar to other joint registries around the world. It was noted that the incidence of avascular necrosis as an indication hip surgery was higher than that seen in registries for developed countries. The mean age for the hip and knee replacement patient in our data was much lower than that what is obtained in the developed world. Furthermore, it was observed that there was increased use of semi-constrained knee prosthesis due to the severe osteoarthritic deformities noted in the patients seen when compared to rate of use of similar implants in the developed world.

**Conclusion:**

This observational study could serve as a springboard for establishment of arthroplasty registries for countries in the sub-region as a whole.

## Introduction

Total hip and knee joint replacement ranks among the most successful surgical interventions and several developing countries in Africa have started to perform these procedures that are routine in developed countries [1]. Both total hip replacement (THR) and total knee replacement (TKR) have been demonstrated to provide long-lasting pain relief and substantial and sustained improvement in joint functioning and health-related quality of life [2]. It is well established as a cost-effective intervention for end-stage lower limb joint disease [3]. In developing countries, success of charity-based trips to developing countries has been heavily reliant on equipment donations and volunteer surgeons, anaesthetists, and ancillary staff including physiotherapists of all grades [4]. Also, it is worth noting that several observational studies have been written about joint arthroplasty in developed communities; however, there is still quite a paucity of knowledge about joint replacement surgery in sub-Saharan Africa. Much has been written about the problems which parts of the developing world face when dealing with the epidemics of infectious diseases, particularly malaria, tuberculosis, and human immunodeficiency virus (HIV) [5]; however, little is still known of the burden of osteoarthritis (OA) in the developing world.

Furthermore, because of large-numbered quality studies and data on joint replacement in the western world, there has been related scientific evaluation which predicts the trend of both replacement and arthroplasty surgery in some of these countries [6], but there are no such studies for joint replacement in the developing world. To the best of our knowledge, there are no studies highlighting the incidence of osteoarthritis in sub-Saharan Africa.

It is also important to note that established literature is not lacking evidence of joint registries in the developed world [7–9], but there is little or no evidence of published arthroplasty registries for developing countries despite the increasing incidence of hip and knee replacements in these countries.

With all of the above literature deficiencies noted, this article is an observational study which seeks to highlight hip and knee arthroplasty work of an orthopaedic charity in a developing country over a period of four years where details on prostheses used were collected including primary and revision cases with the data analysed. It also seeks to help initiate a start off attempt at establishing a joint registry for arthroplasty in Ghana. Furthermore, some trends in demographics, disease pattern, and pathology could also be brought to the fore through this.

## Patients and materials

The following data was prospectively collated from all patients undergoing hip and knee replacements: age, gender, surgical indication/diagnosis, surgeon’s grade (trainee specialist registrar or consultant surgeon—hip and knee arthroplasty surgeon), hospital where surgery was performed, type of anaesthesia used (regional or general), ASA grade (American Society of Anesthesiologists—grades 1 to 5), body mass index (BMI), type of implant (cemented or uncemented) for hip cases and for TKR cases, and the surgical approach (whether semi-constrained, cruciate retaining, or Stanmore prosthesis were used). The type of knee implant bearing was noted (mobile or fixed bearing). Also, the details on the cementing technique used, DVT prophylaxis instituted, antibiotics used, and any intraoperative complications were noted. The above data was compiled from a designed questionnaire and analysis was done. Other supplementary information such as the use of bone grafting and acetabular screws were also included.

### Surgical team and anaesthesia

In 102 THR cases (90%), the primary operating surgeon was an orthopaedic consultant and was assisted by a specialist registrar, while in the remaining 10% of cases, a specialist registrar was the primary operating surgeon with an orthopaedic consultant assisting and supervising. It is worth noting that the latter trend of non-consultant grade as the primary surgeon operating in these cases was seen in the last year of the four year period. For all TKR cases, the primary surgeon was an orthopaedic consultant. In 98% of cases, spinal anaesthesia was the mode of inducing pre-operative anaesthesia for these patients having hip and knee replacement surgery.

### Supplementary information

The majority of the THR carried out were right-sided (68 cases—62.7%) while left-sided was 37.3%. There were only three bilateral cases (2.6%) of the 113 THR cases carried out during this period. All hip replacement cases were carried out via the lateral approach to the hip. Antibiotic prophylaxis treatment was instituted in all cases according to the local hospital antibiotic regime. The deep venous prophylaxis was also prescribed for all cases in the form of subcutaneous clexane once a day until discharge from hospital except where contraindicated. Patients mostly had an ASA grading of 2 and 3. The body mass index was correctly recorded in 74% of THR patients and the mean BMI was 27.5.

## Results

### Total hip replacement

**Fig. 1:**
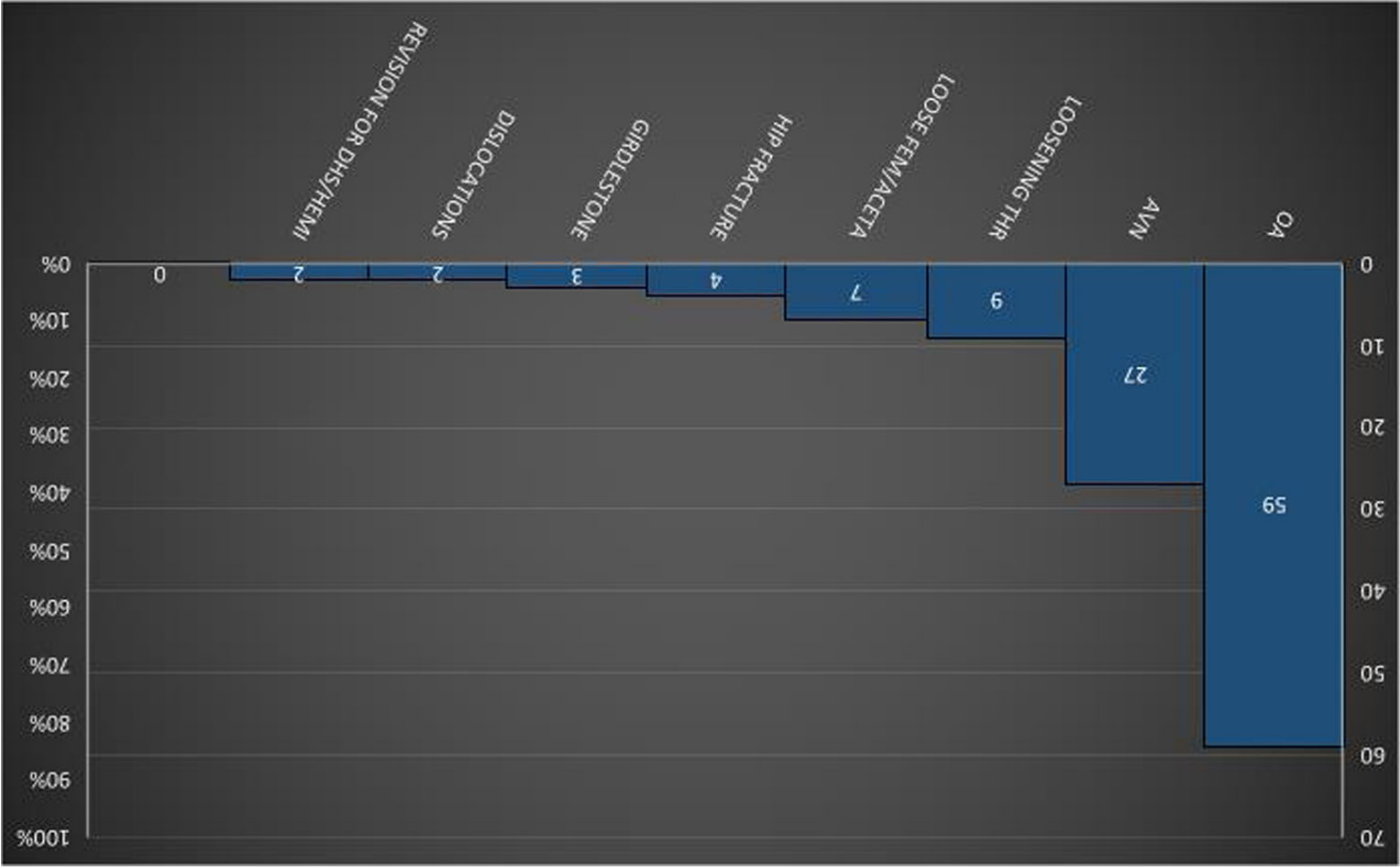
Various indications for total hip replacement in the register period

There were a total of 109 patients which underwent 113 THR procedures. There was an average age of 47.4 years with a range of 17–87 (minimum to maximum). The female-male numbers were 51-62. It was noted that the indication for total hip replacement was primary degenerative osteoarthritis which formed 52.2% of the overall THRs carried out (Fig. 1). Other indications and their percentages were avascular necrosis from sickle cell disease, 23.9% and conversion of girdlestone procedure to THR, 2.7% (Fig. 1)*.*

### Revision arthroplasty

There were 15 (13.2%) revision cases of the hip replacement cases. The indications for the revision included the following: revision for aseptic loosening of implants, 7.9%; loosening of either acetabular or femoral components, 6.2%; and dislocations, 1.8% (Fig. 1).

### Type of total hip replacement

**Fig. 2:**
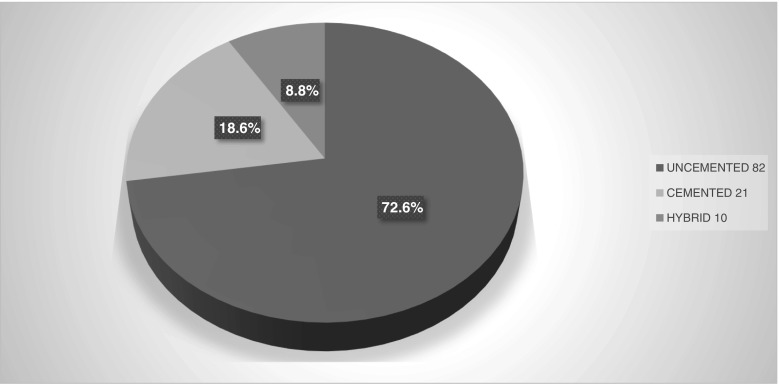
Various types of total hip replacements used in the register period (actual numbers in the legend)

Out of the total number of hip replacement done, 72.6% of them were uncemented, 18.6% cemented, while 8.8% were hybrid cases (Fig. 2).

As regards the articulating surfaces, the ceramic on ceramic bearing surfaces was used for all sickle cell disease patients undergoing hip replacement secondary to avascular necrosis (AVN) which is 23.8% of the total hip replacement cases carried out. Of these sickle cell disease cases, there were seven cases-(25.9%) where controlled osteotomy of the proximal femur was carried out intra-operatively and stabilisation with Dall-Miles cabling systems for insertion of the femoral implants. This was done for very small intramedullary canal sizes of some sickle cell disease patients in order to allow for well-fitting of available femoral components.

Figure 1 shows the commonest osteoarthritic (OA) hip pathology seen in this observational study necessitating the need for total hip replacement procedure carried out as a form of treatment.

### Total knee replacement

**Fig. 3:**
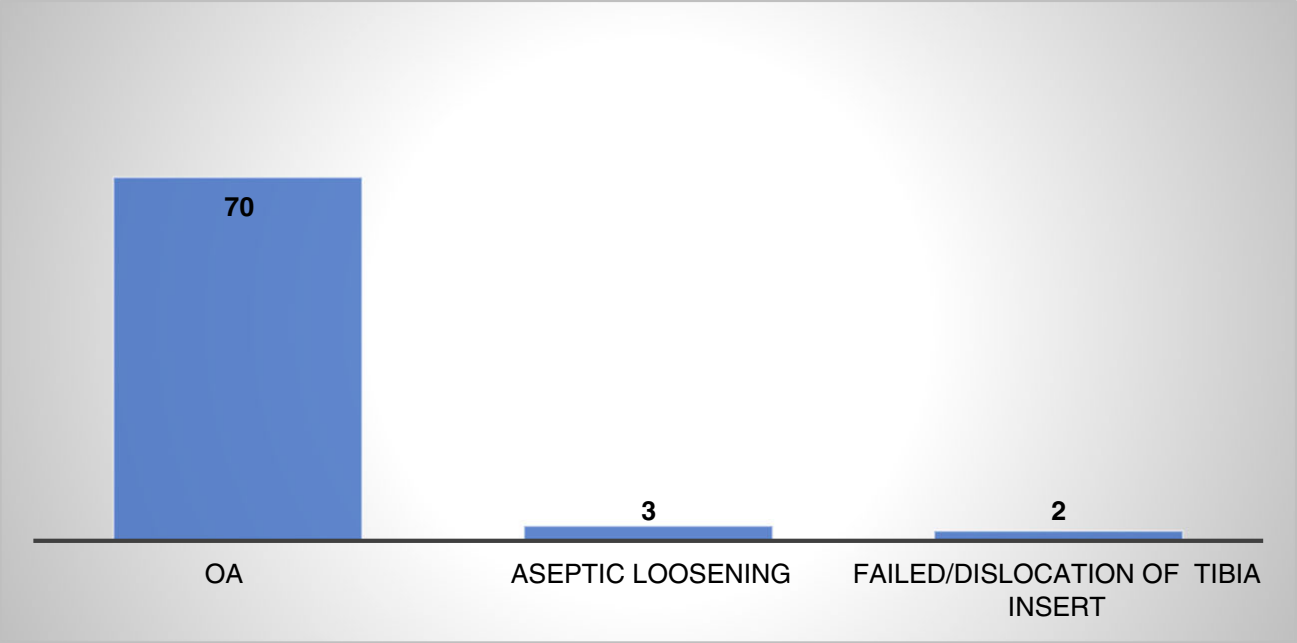
Indications for total knee replacement from the register

There were 82 total knee procedures carried out in 76 patients—the mean age was 60.2 years (range 36–81). The male/female numbers were 26/50. Six patients had bilateral procedures. The indications for TKR were majorly from degenerative osteoarthritis OA (Fig. 3)*.* Other indications include aseptic loosening and failed or dislocated tibial insert. Of all the patients with degenerative OA, it was noted that there was 64 (86.4%) cases with significant valgus deformity while the remainder were varus. Patients who underwent TKR mostly had an ASA grading of 2 and 3. There was however no proper documentation of the BMI in the total knee replacement patients. The parapatellar surgical approach was used in all TKR cases.

### Type of total knee replacement implants

The majority of TKR cases were carried out using the semi-constrained implants for about 62% of cases (see Table 1 for frequency of the other types of TKR implants used). Furthermore, in 23 of cases (28%), patella replacements were done while in 72% of the remaining cases, patella circumcision was carried out instead. As highlighted in Table 1, the semi-constrained total knee replacement was commonly used for the knee OA pathology in the register over the recorded time period—in 62% of cases. The cruciate retaining knee implant was used in only 26% of cases. It is also important to note that in 12% of cases in the knee register, the Stanmore knee prosthesis was used in severe valgus (> 30°) and very significant varus deformity cases, both with significant deficiency in stabilising knee structures—the medial and lateral collateral ligaments. The fixed bearing surfaces for the knee implants were used in the cases where semi-constrained prosthesis was used*.*

**Table 1:** Various types of implants used for the knee degenerative pathologies documented in the register

Type of knee implant	Number (%)
Stanmore hinge knee	10 (12)
Semi-constrained	51 (62)
Cruciate retaining	21 (26)

## Discussion

The data collected in this observational study of joint replacement surgery in Ghana, West Africa, is the introduction to this story of arthroplasty surgery in developing Africa. Lubega et al. published a similar study on the establishment of a national joint registry in Malawi. It was noted that within three years, they had a record of 73 total hip replacement procedures between four centres [10]. In our study, we had 113 hip arthroplasties within four years between two centres. The results are somewhat comparable, but with more centres being involved in our registry, it could increase our numbers significantly. Our study adds to the very few observational work directed at joint registry documentation in developing Africa. This is a step in the right direction but more data is needed to help plot the progression of implant joint arthroplasty in developing West Africa. It was noted that the average age of THR patients in our study was 47.2 years compared to 52 years noted in a Malawian experience. In a previous work by the authors, where they looked at patients who had total knee replacement in Ghana in 2009, it was noted that the mean age of that cohort was 59 years, while in our present observational study of the joint register for total knee replacement between October 2013 and October 2016, the mean age was 60.2 years [10, 11]. Bija et al. [12] also noted in their study into the patterns of knee OA in a hospital in Cameroon that the mean age of patients with OA was 56.9 years. There has been no significant change in the age demographic for the knee replacement patients over different periods. However, the total hip replacement patients also had a difference with a younger age group noted in our study. We could suggest that younger patients are now needing to have hip and knee arthroplasty surgery in developing Africa. The age demographic of younger patients noted in our own register is a huge shift from the older age demographic seen in the developed world. The national joint register for the UK 2017 reports that the mean age of patients having THR procedures was 69.2 years and a similar mean age is noted in a Norwegian joint register report [13, 14]. Furthermore, the mean age is much lower than that seen for TKR patients in developed countries which is about 69.2 years as reported in both the Norwegian and British joint registries.

In our study, it was worth noting that degenerative osteoarthritis is the main indicator for THR; however, avascular necrosis was the second highest indication with 23% incidence in the register. This was seen as a lower percentage when compared to the Malawian study of 47.9% although bearing in mind they had less numbers in their own register [10]. However, when you compare this with the report from a developed country—the UK with 92% of cases—OA was the predominant indication [15]. This shows a different significant pattern in the indication of THR profile. With TKR, the predominant indication was OA and this is a similar trend seen in other TKR joint registries.

**Fig. 4:**
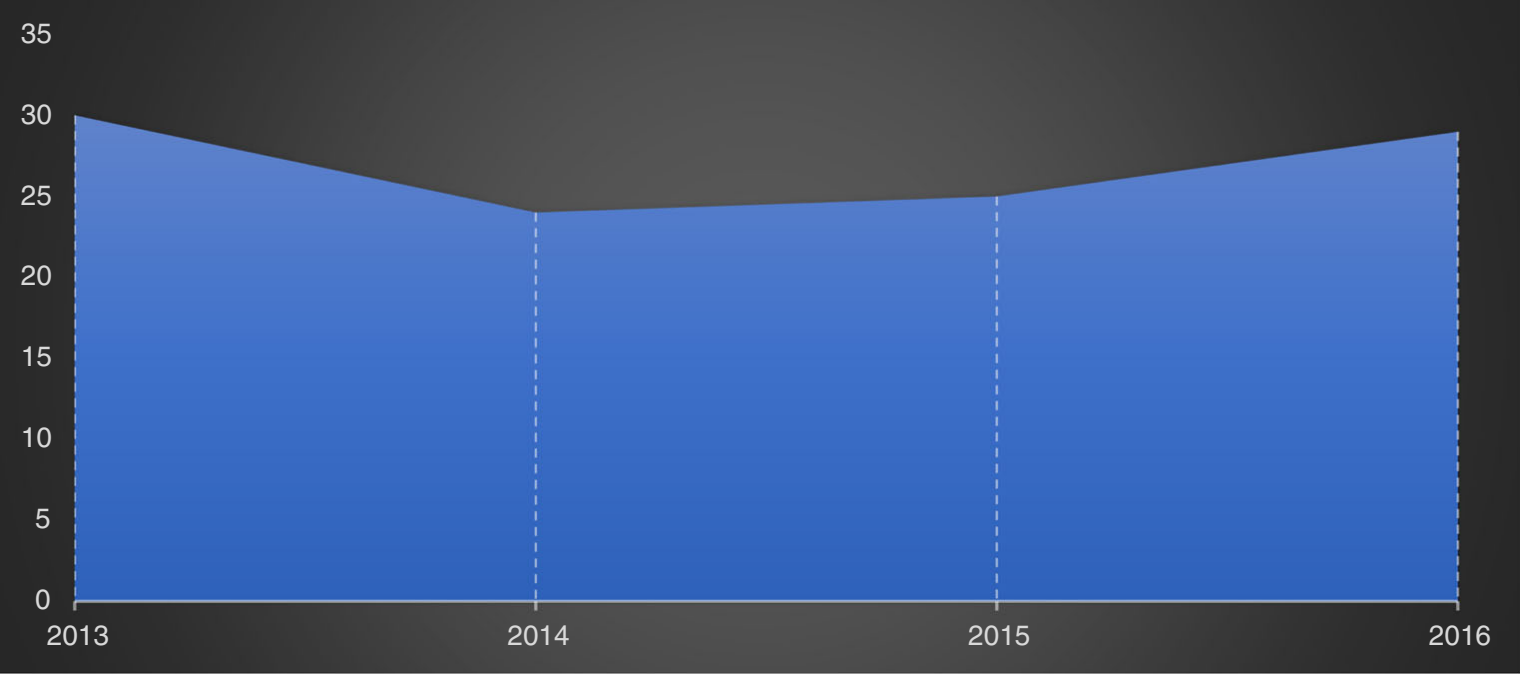
Trendline showing the numbers of THR cases per year

**Fig. 5:**
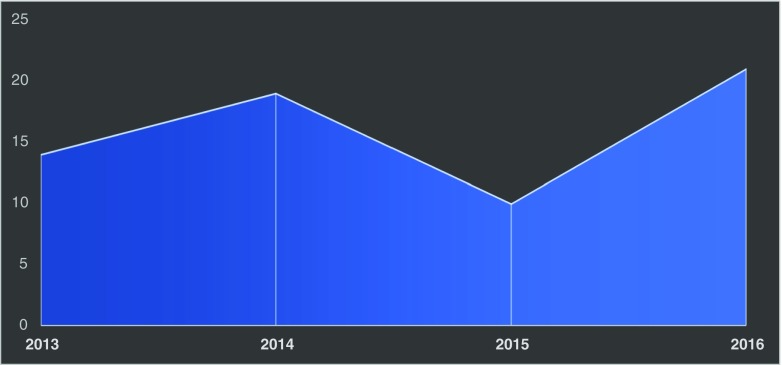
Trendline for number of TKR cases performed per year

It is also important to note the numbers of cases performed and the trend. For total hip replacements, there was a significant increase of cases of about 8% from 2015 to 2016 (Fig. 4) and for knee replacement, an increase of about 30% from 2015 to 2016 (Fig. 5). There was a drop in TKR cases recorded in 2017 (complete data was not available at present). This was due to logistic lapses in taking down adequate records for the cases performed. When this trend is compared to the UK joint registry, there has been a steady rise in cases compared to the numbers seen in our own observational study. There are several reasons for this. Patients in our study still need to pay for joint arthroplasty themselves. In a previous study by the authors, it was noted that patients who underwent TKR procedure had to be able to afford £3500 for perioperative, implants, accessories, and hospital charges [11]. This was still the case for all the patients in our current study who had joint arthroplasty. So cases done largely depended on affordability and so the number trend cannot be predicted and does not show a true representation of the disease burden. The situation is different in the developed world where the national health service and well-funded medical insurance schemes pay for such procedures and rate of joint arthroplasty has been increasing [16]. The lateral approach to the hip was used in all cases in this study which is a contrast to other registries. There is an increase in the use of the posterior approach in a survey carried out among 292 orthopaedic surgeons in 57 countries [17]. The parapatellar approach was used in all cases for total knee replacement in our experience as recorded in the register and this is at per with other knee replacement registries.

Furthermore, in this study, there was increased use in uncemented hip prosthesis with 67.2% rate of use and only about 9% of cases used hybrid implants. The trend in the use of uncemented implants is different currently with increased use of hybrid implants as noted in the UK and Norwegian joint registries reports [14, 15].

Also, for bearing surface type and head size, ceramic on ceramic was mainly used for the sickle cell patients with AVN needing THR who are much younger and the head size 32 is mainly used in our experience with 62% use. This corresponds with similar findings noted in the European registries; however, in the current American joint register, it was noted that the head size 36 was mainly used in 62% of cases [14, 15, 18]. In our own experience, the larger head size 36 mm was used in some patients (when intra-operatively suitable) where it is judged that complying with post-operative instructions will be compromised and hence ensuring hip stability.

For knee replacement arthroplasty surgeries recorded, we note the significantly increased use of semi-constrained implants—used in 62% of cases when compared to the cruciate retaining implants used in only 26% of cases. This is somewhat different from the report of the American and Canadian registries which there was less increased use of constrained implants [18, 19]. This information could give us an idea into the disease pattern of knee OA pathology in these two distinct world regions that is North America and sub-Saharan Africa. The increased use of semi-constrained implants in this study was due to significant valgus arthritic deformity noted in most of knee cases seen*.* This is because most patients here are able to tolerate pain and cope with the deformity before seeking medical help due to financial constraints and this is a similar finding noted in Bija et al.’s [12] study on the knee OA pattern in a hospital setting in sub-Saharan Africa. We also note the low rate of patella replacements in the knee arthroplasty records. We have 28% compared to 66% in the Portuguese arthroplasty [20]. Also, the variation in disease deformity pattern here with 64% of cases having valgus deformity in our register as compared to 20% in Belgian arthroplasty report for 2015–2016 is worth noting [21].

To the best of our knowledge, this article would be the first report at an attempt to highlight joint arthroplasty surgery register in sub-Saharan West Africa. The orthopaedic literature is still devoid of current data as regards hip and knee joint replacement in this region and this article seeks to shed light on the need to encourage more reporting as established in the developed world. National and regional arthroplasty registries have proliferated since the Swedish Knee Arthroplasty Register was started in 1975. Registry reports typically present implant-specific estimates of revision risk and patient- and technique-related factors that can inform clinical decision making about implants and techniques [22]. It would be very informative if a comprehensive and current arthroplasty joint register is established in the developing region of the world. This would add to the body of knowledge of OA disease pattern and could help in future design of implants.

The success and results of THR should be measured by mid- and long-term results and functional results in patients assessed by hip and knee scores [23]. Here in lays the limitation as this is an observation study reporting the incidence of joint arthroplasty in the developing country with no information on functional outcome. Also, recording more information about revision cases could help boost the robustness of the register as seen in other arthroplasty registers.

## Conclusion

This article has highlighted the good work done by an orthopaedic charity in a West African country over four years in bringing the much needed help and treatment to patients requiring hip and knee arthroplasty. It also attempts to start a national joint arthroplasty register which would further help address the information and knowledge deficiency of joint pathology in this part of the world. The awareness and increase of joint arthroplasty surgery expanding in the sub-Saharan region looking ahead the establishment of a comprehensive and current joint register in the countries in this region would go a long way in achieving effective treatment of needy patients and help to reduce the osteoarthritis disease burden.
